# B-type natriuretic peptide (BNP) predicts 90-day mortality and need for paracentesis in cirrhotic patients without systolic heart failure

**DOI:** 10.1038/s41598-020-78946-3

**Published:** 2021-01-18

**Authors:** Tiago Araujo, Ishaan Vohra, Pedro Palacios, Vatsala Katiyar, Estefania Flores, Tejinder Randhawa, Yuchen Wang, Yazan Abu-Omar, Vijaya Mukthinuthalapati, Hemant Mutneja, Sanjay A. Patel, Bashar Attar

**Affiliations:** 1 Department of Internal Medicine, John H. Stroger, Jr. Hospital of Cook County, 1900 West Polk Street, Chicago, IL 60612 USA; 2 Division of Gastroenterology and Hepatology, Department of Medicine, Cook County Health and Hospital System, County, Chicago, IL USA; 3 Department of Medicine, Memorial Sloan Kettering Cancer Center, New York, NY USA

**Keywords:** Gastroenterology, Hepatology, Liver, Liver diseases

## Abstract

Fluid overload is a common complication in patients with cirrhosis. B-type natriuretic peptide (BNP) is a marker of increased blood volume, commonly used in heart failure, that has been shown to be elevated in patients with liver disease. This study examined if BNP levels can be used to determine prognosis and predict worsening of ascites in patients with cirrhosis without concomitant heart disease. A retrospective study was performed at a large urban hospital in Chicago, Illinois and included 430 patients with cirrhosis who had BNP levels ordered during their hospital stay. Patients with clinical heart failure, arrhythmias or pulmonary hypertension were excluded. The primary outcome was 90-day mortality and the secondary outcome was a requirement for therapeutic paracentesis in the 90 days following BNP results. 53 patients (12%) had BNP levels ≥ 300 pg/mL. They had significantly increased serum levels of creatinine, bilirubin, and International Normalized Ratio (INR) when compared to those with BNP < 300 pg/mL. Patients with higher BNP had significantly higher mortality rates (HR 3.49; *p* = 0.037) and were more likely to require therapeutic paracentesis (HR 2.26; *p* = 0.02) in the next 90 days. A BNP ≥ 300 pg/mL had specificity of 88.2% in predicting 90-day mortality. BNP may serve as a practical and reliable marker of underlying disease severity in patients with cirrhosis, with potential to be included in prognostication tools for assessment of end-stage liver disease.

## Introduction

Fluid overload is the most frequent complication of end-stage liver disease, regardless of its etiology^1^. This happens as a low effective circulatory volume from splanchnic vasodilation induces compensatory activation of vasoconstrictor and anti-natriuretic factors, such as the renin–angiotensin–aldosterone system and vasopressin, resulting in water retention^2,3^. More importantly, the degree of fluid accumulation is known to correlate with disease severity in cirrhotic patients, which ultimately led to the inclusion of ascites and hyponatremia in disease-severity prognostication tools^1,4,5^.

Brain natriuretic peptide (BNP) is a 32-amino-acid polypeptide that is produced in response to ventricular stretch in situations of volume or pressure overload^6^. It has gained popularity as an accurate biomarker to support the diagnosis and prognosis of patients with congestive heart failure^7,8^. Recently, increasing attention has also been given to the value of BNP measurement in other clinical settings impacting fluid and hemodynamic values, including renal failure, sepsis, pulmonary hypertension and cirrhosis^9^. The levels of NT-Pro-BNP, Pro-BNP, and BNP have been shown to be elevated in cirrhotic patients when matched to healthy subjects^9–14^. Several small observational studies have also demonstrated a positive correlation between BNP levels and both the Model for End-Stage Liver Disease (MELD) and Child–Pugh scores^11,14–18^. Therefore, it does not come as a surprise that higher natriuretic peptide levels are associated with an increased rate of ascites, spontaneous bacterial peritonitis and other complications^15,19,20^.

To date, there are small observational studies done in Asian and European populations evaluating the role of BNP in predicting mortality of patients with liver disease^14,20–22^. High BNP levels in these subjects can be confounded by concomitant heart failure. Thus, we aimed to investigate if BNP can be used to assess 90-day mortality and paracentesis requirements in cirrhotic patients with no prior history of heart failure.

## Methods

**Figure 1:**
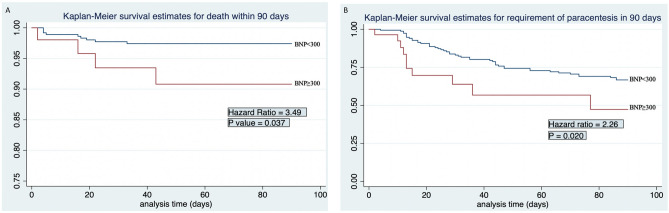
**A** Kaplan–Meier curve comparing 90-day mortality between BNP < 300 and BNP ≥ 300. **B** Kaplan–Meier curve representing 90-day paracentesis requirement based on BNP levels.

We retrospectively reviewed 757 adult patients at an urban safety-net tertiary care center from 2008 to 2018 with a known diagnosis of cirrhosis, confirmed by imaging, and with at least one serum BNP test result in the chart. Exclusion criteria included patients < 18 years of age, a preceding diagnosis of heart failure, documented arrhythmia, pulmonary hypertension and those with incomplete information on study variables. A total of 430 patients met the inclusion criteria (Fig. 1). ARCHITECT BNP assay was used for the BNP analysis, it is a two-step immunoassay for the quantitative measurement of human-BNP in human Ethylenediaminetetraacetic acid (EDTA) plasma using chemiluminescent microparticle immunoassay (CMIA) technology^23,24^.

This study was approved by the Institutional Review Board (IRB) of the Cook County Health & Hospitals System, Chicago. The methods were performed in accordance with the relevant guidelines and regulations set out by IRB, informed consent was obtained from the participants and none of the patients were deceased. The database was set up and maintained by the Department of Medicine, Cook County Health & Hospitals System.

### Study variables

Age, race, viral hepatitis serology, history of alcohol use and medical comorbidities were extracted from each patient’s electronic record. Biochemical data from the date of the first BNP result available was also collected. Child–Pugh and MELD-sodium (MELD-Na) scores were calculated individually based upon available clinical information. Echocardiographic measures (ejection fraction [EF], diastolic dysfunction and left ventricular wall thickness) were documented when available.

### Outcomes

The primary outcome was the rate of 90-day mortality from BNP result date and the secondary outcome was a requirement for therapeutic paracentesis in the subsequent 90 days post-BNP results.

### Statistical analysis

Student’s *t*-test, Wilcoxon rank sum test, or Kruskal–Wallis test was used to compare continuous nonparametric variables and the Chi-square test or Fisher’s exact test was performed to compare categorical variables. Univariable Cox regression analysis was used to calculate unadjusted odds ratios for the primary and secondary outcomes. Survival analysis was performed with time from first BNP to death as the time variable. For the secondary outcome, survival analysis was performed with time from BNP to next paracentesis, excluding paracenteses done within 3 days of BNP results. Patients who did not experience failures were censored at day 90. After univariate screening for all variables with *p* < 0.20, Multivariate Cox regression analysis was performed to evaluate the sensitivity, specificity and likelihood ratios (LR) of BNP for the primary and secondary outcomes. All statistical analyses were performed using STATA (Version 14.0, College station, TX). We considered all variables with *p* < 0.05 to be statistically significant.

## Results

### Patient characteristics

Using a BNP cut-off of 300 pg/mL, we dichotomized our cohort into 2 groups (Table 1); a high BNP group (BNP ≥ 300 pg/mL) and a low BNP group (BNP < 300 pg/mL). 377 (88%) patients had low BNP levels and 53 (12%) patients had high BNP levels. Patients in the high BNP group had lower rates of hepatitis B (0% vs. 40%; *p* = 0.013) and hepatitis C (18.9% vs. 30.2%; *p* = 0.036). They also had significantly increased levels of serum creatinine (2.66 vs 1.03 mg/dL, *p* < 0.001), bilirubin (5.36 vs 2.84 mg/dL, *p* < 0.001), and INR (1.75 vs. 1.50, *p* = 0.003). Echocardiography done within 6 months of BNP results were available for 194 (45%) patients. 188 patients (96.9%) had EF of 55% or greater. No patient had an EF < 45%. Diastolic parameters had been assessed in 117 patients, out of which 70 (59.8%) had no diastolic dysfunction and 39 (33.3%) had grade I diastolic dysfunction (*p* < 0.001. Mean EF for patients in the low and high BNP group was 64% and 60%, respectively (*p* < 0.001). 85% of our patient cohort had a MELD-Na score > 10 and, in these patients, there was significant correlation between MELD-Na and BNP levels (Table 2).

**Table 1:** General characteristics of patients according to BNP values.

Variable	BNP < 300 pg/mL	BNP ≥ 300 pg/mL	*p*-value
Total, n (%)	377 (88)	53 (12)	
Age, years, mean, SD	55.4 (± 10.3)	53.3 (± 12.6)	0.027
Died, n (%)	10 (2.6)	4 (7.5)	0.06
**Race, n (%)**			
African American	93 (24.8)	12 (22.6)	0.748
Hispanic	163 (43.2)	18 (34.0)	0.200
White	80 (21.2)	17 (32.2)	0.077
Asian	12 (3.2)	1 (1.8)	0.606
Other	29 (7.6)	5 (9.4)	0.660
**Clinical characteristics, n (%)**			
Alcohol use	261 (86.7)	40 (13.3)	0.353
Hepatitis B	40 (100)	0 (0)	0.013
Hepatitis C	125 (92.6)	10 (7.4)	0.036
Ascites	168 (85.7)	28 (14.3)	0.258
Diuretic use	107 (82.9)	22 (17.1)	0.051
Number of paracentesis, mean (SD)	1.60 (± 3.25)	2.52 (± 5.1)	0.005
**Laboratory findings, mean (SD)**			
Sodium (mEq/L)	135.16 (± 0.48)	135.98 (± 0.63)	0.558
Creatinine (mg/dL)	1.03 (± 0.96)	2.66 (± 3.25)	< 0.001
Bilirubin (mg/dL)	2.84 (± 4.01)	5.36 (± 8.62)	< 0.001
INR	1.50 (± 0.48)	1.75 (± 0.63)	0.003
**Albumin (mg/dL)**	3.17 (± 6.80)	2.84 (± 0.70)	0.605
Echocardiography findings			
Ejection fraction (%) mean, SD	64.0 (± 0.7)	60.0 (± 1.0)	< 0.001
Grade I diastolic dysfunction (n, %)	70 (59.8%)	39 (33.3%)	< 0.001

*BNP* brain natriuretic peptide, *INR* International Normalized Ratio, *SD* standard deviation.

**Table 2:** Child Pugh Score and MELD-Na predicts prognosis in cirrhotic patients. Child Pugh includes- bilirubin level, albumin, INR, encephalopathy, ascites. MELD Na includes- Bilirubin, dialysis, Creatinine, Sodium, INR. Higher scores correlate with increased mortality.

	BNP < 300 pg/mL	BNP ≥ 300 pg/mL	*p*-value
**Child score**			
A&B	128 (33.9)	12 (22.6)	0.100
C	249 (66.1)	41 (77.4)	
**MELD-Na**			
0–10	59 (15.6)	4 (7.5)	0.118
11–20	189 (50.1)	18 (34.0)	0.027
21–30	94 (24.9)	20 (37.7)	0.048
> 31	35 (9.4)	11 (20.8)	0.011

### Survival analysis

Univariate analysis revealed that, when compared to the low BNP group, patients with higher BNP levels had a significantly higher 90-day mortality rate [HR 3.49 (1.07–11.34); *p* = 0.037] and an increased requirement for paracentesis in the first 90 days from the BNP result [HR 2.26 (1.05–3.79) *p* = 0.02] (Table 3). On multivariate Cox regression analysis, higher BNP levels were associated with a trend towards increased 90-day mortality [HR 1.10 (0.30–3.97); *p* = 0.87] and higher requirements for paracentesis [HR 1.33 (0.63–2.82); *p* = 0.45] (Table 3).

**Table 3:** Univariate and multivariate Cox regression analysis for 90-day Mortality.

Unadjusted odds ratio			Adjusted odds ratio	
Variable	OR (CI)	*p* value	aOR (CI)	*p* value
BNP ≥ 300 pg/dL	3.49 (1.07–11.34)	0.03	1.10 (0.30–3.97)	0.87
Age ≥ 55 years	0.66 (0.22–1.90)	0.44		
≥ 2 paracentesis	1.40 (0.46–4.21)	0.54		
HBV	0.67 (0.08–5.13)	0.70		
HCV	0.34 (0.07–1.54)	0.16	0.65 (0.11–3.67)	0.62
Ascites	2.43 (0.80–7.33)	0.11	1.20 (0.34–4.26)	0.76
Sodium > 135 mEq/L	0.59 (0.20–1.73)	0.34		
Creatinine > 1.22 mg/dL	7.33 (2.45–21.93)	< 0.01	6.77 (1.92–23.86)	< 0.01
Bilirubin > 3.15 mg/dL	5.36 (1.78–16.17)	< 0.01	1.74 (0.41–7.28)	0.44
INR ≥ 1.53	5.92 (1.64–21.31)	0.01	2.56 (0.52–12.66)	0.24
Albumin ≥ 3.12 mg/dL	0.47 (0.13–1.72)	0.26	0.54 (0.12–2.27)	0.40
MELD-Na > 20	6.77 (1.88–24.32)	< 0.01	1.20 (0.22–7.49)	0.76
Race				
African	2.93 (0.82–6.91)	0.10	1.01 (0.08–3.55)	0.99
American	0.32 (0.08–1.17)	0.08	0.46 (0.09–2.39)	0.36
Hispanic	1.02 (0.28–3.69)	0.97	–	–
White	2.67 (0.34–20.55)	0.34	–	–
Asian	1.01 (0.13–7.71)	0.99	–	–
Other				

*CI* confidence interval, *BNP* brain natriuretic peptide, *HBV* hepatitis B, *HCV* hepatitis C.

### Sensitivity, specificity, and likelihood ratios

**Figure 2:**
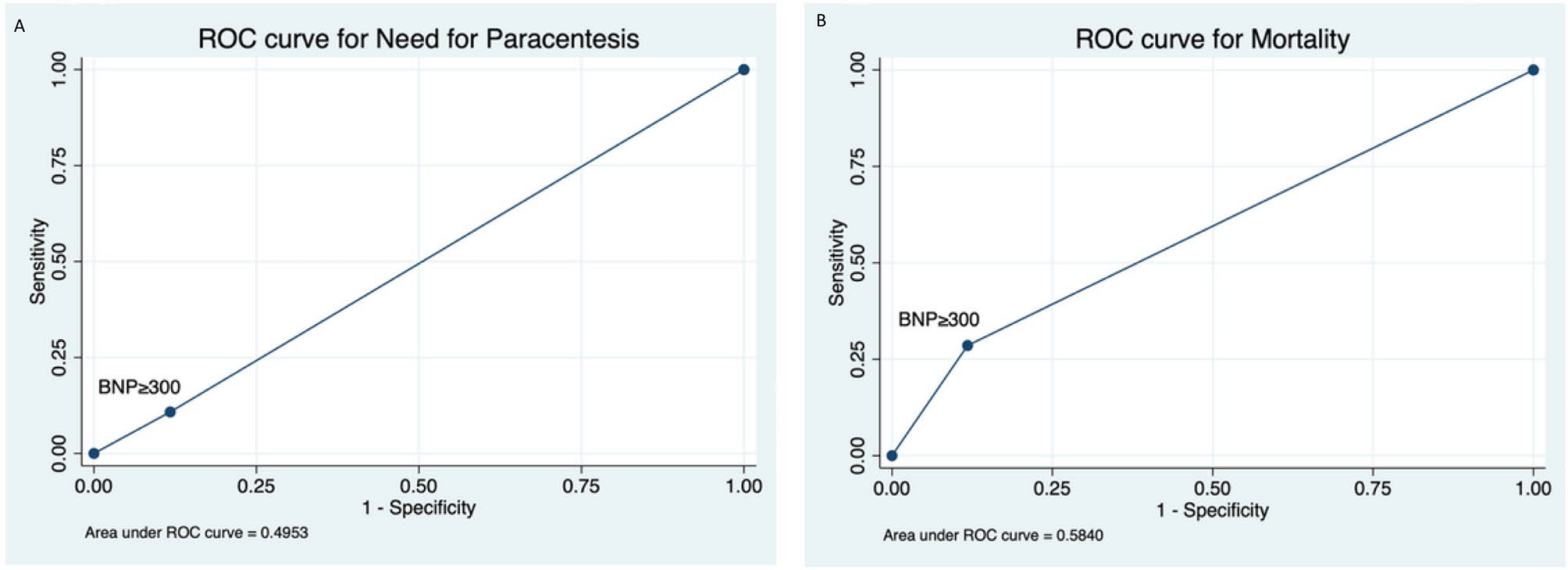
**A** ROC curve representing area under the curve, sensitivity and specificity for mortality based on BNP cut off =  > 300. **B** ROC curve representing area under the curve, sensitivity and specificity for need of paracentesis based on BNP cut off =  > 300.

Secondary analysis to evaluate sensitivity and specificity of BNP ≥ 300 pg/mL for the primary and secondary outcomes was performed. A BNP ≥ 300 pg/mL predicted 90-day mortality with a sensitivity of 28.5% and specificity of 88.2% (positive LR of 2.42; negative LR of 0.80; area under ROC curve 0.58) (Table 4; Figs. 1a, 2a). High BNP levels also predicted a requirement for paracentesis in the following 90 days with a sensitivity of 10.8% and specificity of 88.2% (positive LR 0.92; negative LR 1.0; area under ROC curve 0.49) (Table 4; Figs. 1b, 2b). The sensitivity and specificity of cutoff values are presented in Tables 5 and 6 for mortality and need for paracentesis, respectively.

**Table 4:** Univariate and multivariate Cox Regression Analysis for 90-day requirement of paracentesis.

Unadjusted odds ratio			Adjusted odds ratio	
Variable	OR (CI)	*p* value	aOR (CI)	*p* value
BNP ≥ 300 pg/dL	2.01 (1.05–3.79)	0.03	1.33 (0.63–2.82)	0.45
Age ≥ 55 years	0.94 (0.64–1.38)	0.77		
≥ 2 paracentesis	1.93 (1.20–3.09)	0.01	1.82 (1.12–2.95)	0.01
HBV	0.78 (0.42–1.43)	0.42		
HCV	1.05 (0.69–1.58)	0.81		
Ascites	1.00	–		
Sodium > 135 mEq/L	0.95 (0.64–1.40)	0.82		
Creatinine > 1.22 mg/dL	2.18 (1.35–3.52)	< 0.01	1.97 (1.12–2.95)	0.01
Bilirubin > 3.15 mg/dL	1.99 (1.29–3.06)	< 0.01	1.39 (0.82–2.34)	0.21
INR ≥ 1.53	1.55 (1.05–2.31)	0.02	1.31 (0.79–2.16)	0.29
Albumin ≥ 3.12 mg/dL	0.54 (0.33–0.87)	0.01	0.60 (0.36–1.02)	0.06
Child–Pugh Class C	1.89 (1.25–2.87)	< 0.01	1.00	–
MELD-Na > 20	1.67 (1.14–2.49)	0.01	0.84 (0.49–1.44)	0.54
Race				
African	1.30 (0.83–2.05)	0.24		
American	0.79 (0.52–1.18)	0.25		
Hispanic	1.05 (0.69–1.60)	0.81		
White	2.04 (0.50–8.37)	0.31		
Asian	0.89 (0.46–1.73)	0.74		
Other				

*CI* confidence interval, *BNP* brain natriuretic peptide, *HBV* hepatitis B, *HCV* hepatitis C.

**Table 5:** Sensitivity and specificity for BNP to predict 90-day mortality.

Value of BNP (pg/mL)	Sensitivity (%)	Specificity (%)	LR +	LR −
0–100	100	0.00	1.00	–
300	28.5	88.2	2.42	0.80
400	28.5	92.0	3.60	0.77
600	28.5	93.9	4.75	0.76
1000	21.4	98.3	12.7	0.79

**Table 6:** Sensitivity and specificity for BNP to predict 90-day mortality.

Value of BNP (pg/mL)	Sensitivity (%)	Specificity (%)	LR +	LR −
0–100	100	0.00	1.00	–
300	10.8	88.2	0.92	1.01
400	7.21	91.9	0.89	1.01
600	4.50	93.5	0.69	1.02
1000	0.90	97.5	0.37	1.02

## Discussion

This is the largest known study assesssing BNP as a prognostication tool in patients with cirrhosis. Despite excluding patients with overt cardiac dysfunction, we found a significant correlation of BNP levels with medium-term mortality and paracentesis requirements.

Several studies have demonstrated elevated BNP levels in individuals with cirrhosis when compared to those with non-alcoholic fatty liver disease and healthy controls, although at a lower magnitude than that usually seen in heart failure^10–14,21,25^. The underlying cause is often attributed to subclinical heart disease or overt cirrhotic cardiomyopathy, with higher levels seemingly correlated with greater degrees of systolic dysfunction^18,21^. Other proposed mechanisms include decreased renal clearance and altered hepatic degradation^14^, although this has been subject of debate. Henriksen et al. found that both the rate of pro-BNP cleavage and the hepatic disposal of pro-BNP and BNP were not significantly different in cirrhotic patients and controls^11^. It has also been hypothesized that the hyperdynamic circulation pattern often seen in cirrhotic patients could potentially trigger BNP release, but recent studies show an inverse correlation between a hyperdynamic state and BNP levels^11,21^.

In our cohort, patients with a known diagnosis of heart failure or arrhythmias were excluded to minimize confounding factors. The increased BNP levels among included patients would, therefore, signal either a very early stage of cardiac involvement or point to other mechanisms for BNP elevation, such as decreased renal clearance. This is supported by higher BNP levels in our patients with creatinine levels above the median. It has also been proposed that BNP and Pro-BNP function as markers of very early stage heart disease in cirrhotic patients, and abnormal levels may precede clinical heart failure or echocardiographic changes^15,16,26^.

When using a cut-off of 300 pg/mL, BNP predicted 90-day all-cause mortality. These results come in agreement with previous studies done in cirrhotic patients. Shi et al. found that a BNP > 167 pg/mL was an independent predictor of disease progression and 12-month mortality^14^. Zhao et al. concluded that pro-BNP correlated with disease severity and in-hospital mortality regardless of their systolic function^20^. The association of natriuretic peptides and mortality has also been studied in the setting of liver transplant to predict post-transplant mortality in patients with cirrhosis, where levels of pre-operative BNP < 155 pg/mL had excellent negative predictive value for ICU mortality, even in the context of high MELD scores^22^.

BNP levels seem to correlate with Child–Pugh and MELD scores in several small observational studies^11,14–18^. We found a significant association between BNP levels and MELD-Na scores and a trend towards correlation with the Child–Pugh stratification system (*p* = 0.01). Pimenta et al. had similar findings and proposed that the homogeneity of their patients, who often had severe liver disease, contributed to other parameters having a greater influence on BNP levels^21^. Similarly, our cohort consisted primarily of patients with Child C cirrhosis (72.7%). Clinical differences among individuals in this subgroup were likely more critical in determining BNP levels.

Higher levels of natriuretic peptides in patients with ascites have been reported in the literature^14,15,19,20,24^ and the role of BNP and NT-Pro-BNP as part of the initial work-up of ascites has also been studied^25,28^. Patients with severe cirrhosis undergo paracentesis more often, and the procedure itself has been shown to temporarily improve echocardiographic parameters and decrease mortality^19,27^. Therefore, we hypothesized that the degree of BNP elevation could serve as a surrogate marker of ascites severity, potentially predicting the timeframe at which patients would require their next therapeutic paracentesis. Using a cut-off of 300 pg/mL, BNP predicted the need for paracentesis in the next 90 days. BNP levels could potentially be implemented in protocols assessing patients at risk for early readmission who could benefit from scheduled outpatient procedures.

### Limitations

This study has limitations inherent to its retrospective design. The reason for ordering BNP levels was not routinely documented and likely implies patients had symptoms concerning for fluid overload of cardiac origin at presentation. Patients who were fully asymptomatic were unlikely to be included, as they were not likely to visit the emergency department nor have serum BNP levels drawn. Several patients had kidney dysfunction, which may have contributed to elevated BNP. However, kidney function tests also reflect the severity of cirrhosis and are used in prognostication tools, thus these patients could not be excluded from the cohort. Patients with undiagnosed, asymptomatic paroxysms or non-sustained arrhythmia could not be entirely excluded. The small numbers of echocardiogram available within 90 days (20%) and 6 months (45%) was multifactorial. Possible reasons include: (1) lack of clinical suspicion of heart failure, (2) scarcity of resources in the public health system, (3) a baseline patient population with notable issues surrounding social determinants of health, including substance abuse, mental health issues, immigrant status, lack of insurance and transportation. The number of heart failure patients at 90 days and 6 months were zero in our cohort as per inclusion criteria. Lastly, ideal body/dry weight was not recorded. Even though there are studies correlating obesity with lower BNP levels, there was no practical way to differentiate obesity from fluid accumulation impacting the weight measurement of these patients.

## Conclusion

BNP may serve as a surrogate marker for disease severity and symptomatic burden in patients with cirrhosis. Prospective studies are needed to assess its inclusion into prognostication tools for end-stage liver disease and early identification of patients with higher mortality and paracentesis requirements.
